# Efficient Biexciton State Preparation in a Semiconductor Quantum Dot Coupled to a Metal Nanoparticle with Linearly Chirped Gaussian Pulses

**DOI:** 10.3390/nano12183098

**Published:** 2022-09-07

**Authors:** Athanasios Smponias, Dionisis Stefanatos, George P. Katsoulis, Ioannis Thanopulos, Emmanuel Paspalakis

**Affiliations:** 1 Materials Science Department, School of Natural Sciences, University of Patras, 26504 Patras, Greece; 2 Department of Physics and Astronomy, University College London, Gower Street, London WC1E 6BT, UK

**Keywords:** semiconductor quantum dots, biexciton, coherent control, adiabatic rapid passage

## Abstract

We consider a hybrid nanostructure composed of a semiconductor quantum dot placed near a spherical metallic nanoparticle, and study the effect of the nanoparticle on the population transferral from the ground to the biexciton state of the quantum dot, when using linearly chirped Gaussian pulses. For various values of the system parameters (biexciton energy shift, pulse area and chirp, interparticle distance), we calculate the final population of the biexciton state by performing numerical simulations of the non-linear density matrix equations which describe the coupled system, as well as its interaction with the applied electromagnetic field. We find that for relatively large values of the biexciton energy shift and not very small interparticle distances, the presence of the nanoparticle improves the biexciton state preparation, since it effectively increases the area of the applied pulse. For smaller biexciton energy shifts and smaller distances between the quantum dot and the nanoparticle, the performance is, in general, degraded. However, even in these cases we can still find ranges of parameter values where the population transfer to the biexciton state is accomplished with high fidelity, when using linearly chirped Gaussian pulses. We anticipate that our results may be exploited for the implementation of novel nanoscale photonic devices or future quantum technologies.

## 1. Introduction

Manipulating the exciton and biexciton states in semiconductor quantum dots using laser fields is an intense research field, because such systems provide a promising solid state platform for modern quantum technologies [1]. Within this framework, several works study the optical properties of hybrid systems composed of semiconductor quantum dots coupled to plasmonic nanostructures [2,3]. By coherently controlling the quantum part of such composite nanosystems, they function as active nanophotonic structures which are anticipated to find major applications in areas, such as nanotechnology and quantum technology. As an example we mention that a composite structure consisting of a semiconductor quantum dot (SQD) and a metal nanoparticle (MNP), is more efficient than a quantum dot alone for optical phenomena, such as the creation of single photons on demand [4,5] and polarization-entangled photons [6]. In order to take advantage of the superior properties provided by the SQD-MNP system regarding these quantum technological applications, an important challenge is to efficiently prepare the biexciton state starting initially from the grounds state of the quantum dot, when the nanoparticle is present. We note that significant work on the efficient preparation and manipulation of the biexciton state in a SQD with applications in quantum technology has been performed in the absence of the MNP [7,8,9,10,11,12,13,14,15,16,17].

In our recent works, we have tackled the problem of efficient generation of the biexciton state of a SQD-MNP coupled structure using resonant hyperbolic secant [18] and on–off pulses [19], as well as pulses designed using the methodology of shortcuts to adiabaticity [20]. Although these methods appear to be successful in theory, they may present some problems in the experimental implementation. Specifically, the resonant pulses might not have the necessary robustness against unexpected frequency detunings, while the shortcut pulse profiles might be difficult to implement experimentally. For these reasons, here we investigate the problem of biexciton state preparation in the SQD-MNP hybrid structure using easily implementable linearly chirped Gaussian pulses, and explore rapid adiabatic passage for the efficient preparation of the biexciton state [21,22]. Note that such pulses have been successfully used for population transfer to the biexciton state in a single quantum dot [23,24], without the MNP, while here we study the problem in the presence of the MNP. In addition, very recently chirped Gaussian pulses have been applied to efficiently generate the exciton state in a coupled SQD-MNP structure [25]. We find with numerical simulations that the desired population transfer can be implemented with the used pulses. We also find that for large biexciton energies the nanoparticle improves the robustness of the population transfer, while for moderate to small biexciton energies its presence reduces the efficiency. However, even in the latter cases we can still find ranges of parameters where the population transfer is successfully accomplished.

This article is structured as follows. In Section 2 we discuss the system under study and in Section 3 the applied pulses. In Section 4 we present the results from numerical simulations of the system, and in Section 5 we summarize our findings.

## 2. System

**Figure 1:**
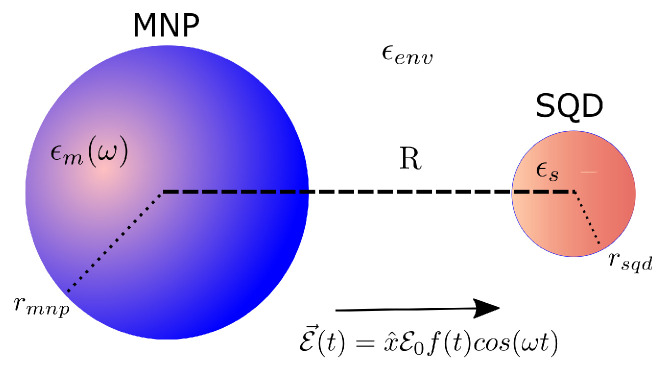
Semiconductor quantum dot–metal nanoparticle coupled system.

The coupled SQD-MNP system is displayed in Figure 1. The Hamiltonian of this system, in the dipole approximation, can be expressed as [18,19,20]

(1) $$H=E_{1}\left|1\rangle \langle 1\right|+\left(2E_{1}+E_{b}\right)\left|2\rangle \langle 2\right|-\mu E_{\mathrm{SQD}}\left(t\right)\left(|0\rangle \langle 1\right|+\left|1\rangle \langle 2\right|+H.c.)\;.$$

In the above expression, $E_{1}$ is the energy of the single-exciton state |1〉 and $E_{b}$ the energy shift of the biexciton state |2〉. For simplicity, we have taken the energy of the ground state |0〉 to be the zero of the energy. Additionally, μ denotes the dipole moment of the SQD corresponding to the ground-exciton transition and the exciton-biexciton transition (in order to simplify things, this is taken the same for both transitions), and $E_{\mathrm{SQD}}$ represents the electric field inside the SQD. We emphasize that we consider a symmetric quantum dot and because of the selection rules there is no direct ground to biexciton transition with a single photon.

In the dipole approximation, the total electric field inside the quantum dot consists of two parts, where one part is due to the applied external field and the other part to the induced field produced by the polarization of the metal nanoparticle (taken into account as a classical metallic nanosphere). We assume that the system interacts with a linearly polarized electric field with $E\left(t\right)=E_{0}f\left(t\right)\cos\left[\omega t+\phi \left(t\right)\right]$, that excites both the ground-exciton and the exciton–biexciton transitions in the semiconductor quantum dot. Here, $E_{0}$ is the electric field amplitude, f(t) is the dimensionless pulse envelope, ω is the angular frequency, and ϕ(t) is the time-dependent phase. Actually, in order to properly calculate $E_{QSD}$ we have to separate the positive and negative frequency contributions since they exhibit different time response. Then, $E_{\mathrm{SQD}}$ is explicitly written as [26,27,28,29,30]:

(2) $$E_{\mathrm{SQD}}\left(t\right)=\frac{\hbar }{\mu }\left\{\frac{\Omega (t)}{2}+G\left[\sigma_{10}\left(t\right)+\sigma_{21}\left(t\right)\right]\right\}e^{-i[\omega t+\phi (t)]}+H.c.$$

In this equation, we introduced the slowly varying quantities $\sigma_{21}\left(t\right)=\rho_{21}\left(t\right)e^{i[\omega t+\phi (t)]}$ and $\sigma_{10}\left(t\right)=\rho_{10}\left(t\right)e^{i[\omega t+\phi (t)]}$, where $\rho_{ij}\left(t\right)$ are the density matrix elements. We also defined the time-dependent Rabi frequency Ω(t) as [26,27,28,29]

(3) $$\Omega \left(t\right)=\Omega_{0}f\left(t\right)\;,\;\Omega_{0}=\frac{\mu E_{0}}{\hbar \varepsilon_{effS}}\left(1+\frac{s_{a}\gamma_{1}r_{mnp}^{3}}{R^{3}}\right)\;,$$

and parameter *G* as [27]

(4) $$G=\sum_{n=1}^{N}\frac{1}{4\pi \varepsilon_{env}}\frac{{\left(n+1\right)}^{2}\gamma_{n}r_{mnp}^{2n+1}\mu^{2}}{\hbar \varepsilon_{effS}^{2}R^{2n+4}}\;.$$

Here, $\varepsilon_{effS}=\frac{2\varepsilon_{env}+\varepsilon_{S}}{3\varepsilon_{env}}$, $\gamma_{n}=\frac{\varepsilon_{m}\left(\omega \right)-\varepsilon_{env}}{\varepsilon_{m}\left(\omega \right)+\left(n+1\right)\varepsilon_{env}/n}$ with n=1,2,3,⋯, where $\varepsilon_{S},\varepsilon_{m},\varepsilon_{env}$ express the dielectric constants of SQD, MNP and the environment, respectively, and $s_{a}=2$ as the applied field is taken parallel to the interparticle axis of the system. *R* is the SQD-MNP distance and $r_{mnp}$ is the MNP radius.

The time-dependent Rabi frequency contains two terms, one related to the direct coupling of the quantum dot to the applied field, and another related to the electric field from the metal nanoparticle which is induced by the external field. In addition, parameter *G* emerges because of the electromagnetic interactions between excitons and plasmons [26,27,31]. This self-interaction term has its origin in the induced dipole on the metal nanoparticle, that is produced by the dipole induced by the applied field on the semiconductor quantum dot [26,28,31]. The formula of Equation (4) accounts for multipole effects and provides a higher accuracy for *G* [27]. In the subsequent calculation we use N=20, an adequate value in order to achieve convergence.

Using Hamiltonian (1) and following the theory of density matrix dynamics, in the rotating wave approximation, we obtain the following equations for the slowly varying envelopes of the density matrix elements

(5) $$\begin{array}{ccc} {\dot{\sigma }}_{00}\left(t\right) & = & \Gamma_{11}\sigma_{11}\left(t\right)+i\frac{\Omega^{*}\left(t\right)}{2}\sigma_{10}\left(t\right)-i\frac{\Omega (t)}{2}\sigma_{01}\left(t\right)\\ & + & iG^{*}\left[\sigma_{01}\left(t\right)+\sigma_{12}\left(t\right)\right]\sigma_{10}\left(t\right)-iG\left[\sigma_{10}\left(t\right)+\sigma_{21}\left(t\right)\right]\sigma_{01}\left(t\right)\;, \end{array}$$

(6) $$\begin{array}{ccc} {\dot{\sigma }}_{22}\left(t\right) & = & -\Gamma_{22}\sigma_{22}\left(t\right)+i\frac{\Omega (t)}{2}\sigma_{12}\left(t\right)-i\frac{\Omega^{*}\left(t\right)}{2}\sigma_{21}\left(t\right)\\ & + & iG\left[\sigma_{10}\left(t\right)+\sigma_{21}\left(t\right)\right]\sigma_{12}\left(t\right)-iG^{*}\left[\sigma_{01}\left(t\right)+\sigma_{12}\left(t\right)\right]\sigma_{21}\left(t\right)\;, \end{array}$$

(7) $$\begin{array}{ccc} {\dot{\sigma }}_{01}\left(t\right) & = & \left[i\frac{E_{1}}{\hbar }-i\left(\omega +\dot{\phi }\right)-\gamma_{01}\right]\sigma_{01}\left(t\right)+i\frac{\Omega^{*}\left(t\right)}{2}\left[\sigma_{11}\left(t\right)-\sigma_{00}\left(t\right)\right]-i\frac{\Omega (t)}{2}\sigma_{02}\left(t\right)\\ & + & iG^{*}\left[\sigma_{01}\left(t\right)+\sigma_{12}\left(t\right)\right]\left[\sigma_{11}\left(t\right)-\sigma_{00}\left(t\right)\right]-iG\left[\sigma_{10}\left(t\right)+\sigma_{21}\left(t\right)\right]\sigma_{02}\left(t\right)\;, \end{array}$$

(8) $$\begin{array}{ccc} {\dot{\sigma }}_{02}\left(t\right) & = & \left[i\frac{2E_{1}+E_{b}}{\hbar }-2i\left(\omega +\dot{\phi }\right)-\gamma_{02}\right]\sigma_{02}\left(t\right)+i\frac{\Omega^{*}\left(t\right)}{2}\left[\sigma_{12}\left(t\right)-\sigma_{01}\left(t\right)\right]\\ & + & iG^{*}\left[\sigma_{12}^{2}\left(t\right)-\sigma_{01}^{2}\left(t\right)\right]\;, \end{array}$$

(9) $$\begin{array}{ccc} {\dot{\sigma }}_{12}\left(t\right) & = & \left[i\frac{E_{1}+E_{b}}{\hbar }-i\left(\omega +\dot{\phi }\right)-\gamma_{12}\right]\sigma_{12}\left(t\right)+i\frac{\Omega^{*}\left(t\right)}{2}\left[\sigma_{22}\left(t\right)-\sigma_{11}\left(t\right)\right]+i\frac{\Omega (t)}{2}\sigma_{02}\left(t\right)\\ & + & iG^{*}\left[\sigma_{01}\left(t\right)+\sigma_{12}\left(t\right)\right]\left[\sigma_{22}\left(t\right)-\sigma_{11}\left(t\right)\right]+iG\left[\sigma_{10}\left(t\right)+\sigma_{21}\left(t\right)\right]\sigma_{02}\left(t\right)\;. \end{array}$$

In the above equations $\Gamma_{11}$, $\Gamma_{22}$ denote the decay rates of the single-exciton and biexciton states, respectively, and $\gamma_{nm}$, with n≠m the dephasing rates of the system. In addition, $\sigma_{nn}\left(t\right)=\rho_{nn}\left(t\right)$, consequently $\sigma_{00}\left(t\right)+\sigma_{11}\left(t\right)+\sigma_{22}\left(t\right)=1$, and $\sigma_{01}\left(t\right)=\rho_{01}\left(t\right)e^{-i[\omega t+\phi (t)]}$, $\sigma_{02}\left(t\right)=\rho_{02}\left(t\right)e^{-2i[\omega t+\phi (t)]}$, and $\sigma_{12}\left(t\right)=\rho_{12}\left(t\right)e^{-i[\omega t+\phi (t)]}$. In the following, we also set the laser frequency to the two-photon resonance value

(10) $$\hbar \omega =E_{1}+\frac{E_{b}}{2}.$$

## 3. Methods

Using Equations (5)–(9) with $\Gamma_{11}=\Gamma_{22}=\gamma_{01}=\gamma_{12}=\gamma_{02}=0$ and $E_{b}=0$ for the variables

(11) $$\begin{array}{ccc} \Delta (t) & = & \sigma_{00}\left(t\right)-\sigma_{22}\left(t\right), \end{array}$$

(12) $$\begin{array}{ccc} \sigma (t) & = & \frac{1}{\sqrt{2}}\left[\sigma_{10}\left(t\right)+\sigma_{21}\left(t\right)\right], \end{array}$$

we obtain the equations

(13) $$\begin{array}{ccc} \dot{\Delta }\left(t\right) & = & i{\tilde{\Omega }}^{*}\left(t\right)\sigma \left(t\right)-i\tilde{\Omega }\left(t\right)\sigma^{*}\left(t\right)+4G_{I}\sigma \left(t\right)\sigma^{*}\left(t\right), \end{array}$$

(14) $$\begin{array}{ccc} \dot{\sigma }\left(t\right) & = & i\dot{\phi }\left(t\right)\sigma \left(t\right)+i\frac{\tilde{\Omega }\left(t\right)}{2}\Delta \left(t\right)+iG\Delta \left(t\right)\sigma \left(t\right), \end{array}$$

where $\tilde{\Omega }\left(t\right)=\Omega \left(t\right)/\sqrt{2}=\left|\Omega \left(t\right)\right|e^{i\beta }/\sqrt{2}$ and GI=Im{G}. Note that at t=0 the initial conditions corresponding to the ground state are Δ(0)=1,σ(0)=0, and perfect biexciton preparation at the final time $t=t_{f}$ corresponds to the target value $\Delta (t_{f})=-1$.

Now observe that for G=0 the above equations become the two-level system equations

(15) $$i\left(\begin{array}{c} \dot{a_{1}}\left(t\right)\\ \dot{a_{2}}\left(t\right) \end{array}\right)=\frac{1}{2}\left(\begin{array}{cc} -\dot{\phi }\left(t\right) & \tilde{\Omega }\left(t\right)\\ {\tilde{\Omega }}^{*}\left(t\right) & \dot{\phi }\left(t\right) \end{array}\right)\left(\begin{array}{c} a_{1}\left(t\right)\\ a_{2}\left(t\right) \end{array}\right),$$

with the correspondence $\Delta \left(t\right)=|a_{1}{\left(t\right)|}^{2}-{\left|a_{2}\left(t\right)\right|}^{2}$ and $\sigma \left(t\right)=a_{1}\left(t\right)a_{2}^{*}\left(t\right)$. Thus, the biexciton state preparation from Δ(0)=1 to $\Delta (t_{f})=-1$ is equivalent to inverting the population in this two-level system. The instantaneous eigenstates and eigenvalues of the two-level system are

(16) $$\begin{array}{ccc} |\psi_{+}\left(t\right)\rangle & = & \left(\begin{array}{c} \cos\frac{\theta (t)}{2}\\ \sin\frac{\theta (t)}{2}e^{-i\beta } \end{array}\right), \end{array}$$

(17) $$\begin{array}{ccc} |\psi_{-}\left(t\right)\rangle & = & \left(\begin{array}{c} \sin\frac{\theta (t)}{2}\\ -\cos\frac{\theta (t)}{2}e^{-i\beta } \end{array}\right) \end{array}$$

and

(18) $$A_{\pm }\left(t\right)=\pm \frac{1}{2}\sqrt{{\dot{\phi }}^{2}\left(t\right)+{\left|\tilde{\Omega }\left(t\right)\right|}^{2}}\;,$$

where

(19) $$\tan\theta (t)=\frac{|\tilde{\Omega }\left(t\right)|}{-\dot{\phi }\left(t\right)}$$

while recall that $\tilde{\Omega }\left(t\right)=\Omega \left(t\right)/\sqrt{2}=\left|\Omega \left(t\right)\right|e^{i\beta }/\sqrt{2}$. If the applied field is chosen so the mixing angle changes slowly from θ(0)=0 to $\theta (t_{f})=\pi$, then the population inversion occurs adiabatically following the eigenstate $|\psi_{+}\left(t\right)\rangle$. On the other hand, if θ(0)=π is slowly changed to $\theta (t_{f})=0$, the inversion takes place following the eigenstate $|\psi_{-}\left(t\right)\rangle$.

To accomplish the targeted population inversion and the corresponding biexciton state preparation, we will employ linearly chirped Gaussian pulses. We explain briefly how such a pulse can be obtained when starting from a pulse with constant frequency and Gaussian profile

(20) $$f\left(t\right)=\exp\left[-\frac{{\left(t-t_{0}\right)}^{2}}{2\tau_{0}^{2}}\right],$$

i.e.,

(21) $$E=E_{0}\exp\left[-\frac{{\left(t-t_{0}\right)}^{2}}{2\tau_{0}^{2}}\right]\cos\omega t,$$

where

(22) $$E_{0}=\frac{\hbar \epsilon_{effs}}{\mu }\frac{\Theta }{\sqrt{2\pi }\tau_{0}}$$

is the amplitude and

(23) $$\Theta =\int_{-\infty }^{\infty }f\left(t\right)dt,$$

is the pulse area, which equals $\sqrt{2\pi }\tau_{0}$ for the profile (20). If the pulse (21) passes through a chirp filter characterized by a chirp constant *a*, it is transformed to the pulse [23,32]

(24) $$E\left(t\right)=\frac{\hbar \epsilon_{effs}}{\mu }\frac{\Theta }{\sqrt{2\pi \tau_{0}t_{p}}}\exp\left[-\frac{{\left(t-t_{0}\right)}^{2}}{2t_{p}^{2}}\right]\cos\left(\omega t+\phi (t)\right),$$

where its duration is modified from $\tau_{0}$ to [23,32]

(25) $$t_{p}=\sqrt{\tau_{0}^{2}+\frac{a^{2}}{\tau_{0}^{2}}}\;,$$

and its frequency obtains a linear chirp

(26) $$\dot{\phi }\left(t\right)=c\left(t-t_{0}\right),$$

with chirp rate [23,32]

(27) $$c=\frac{a}{a^{2}+\tau_{0}^{4}}.$$

The pulse duration is set to $t_{f}=2t_{0}$ with long enough $t_{0}$, which also determines the pulse center.

Note that for c>0(a>0) the mixing angle varies from 0 to π, thus the system follows $|\psi_{+}\left(t\right)\rangle$, while for c<0(a<0) it varies from π to 0 and the system follows $|\psi_{-}\left(t\right)\rangle$.

## 4. Results and Discussion

We numerically simulate Equations (5)–(9) with the parameter values: $\Gamma_{11}^{-1}=\Gamma_{22}^{-1}\;=0.8\;$ ns, $\gamma_{01}^{-1}=\gamma_{02}^{-1}=\gamma_{12}^{-1}\;=0.3\;$ ns, $\varepsilon_{env}=\varepsilon_{0}$, $\varepsilon_{s}=6\varepsilon_{0}$, $E_{1}=2.5\;$ eV, μ=0.65*e*nm, and $r_{mnp}=7.5\;$ nm, with $\varepsilon_{0}$ denoting the vacuum dielectric constant. These values have been utilized in many studies of the systems at hand, see, for example, Refs. [18,19,20], and represent typical values for CdSe-based quantum dots. The reason behind choosing CdSe-based quantum dots is that the localized surface plasmon has the main contribution near the exciton energy of the quantum dot, as it has a plasmon resonance near that frequency. The results would be analogous for other quantum dot structures, f.e., GaAs-based or InAs/GaAs, which nevertheless have much smaller exciton energies, thus the influence of the nanoparticle is much smaller since they are away from the plasmon resonance frequency. An example of coherent control in a SQD-MNP coupled system involving a CdSe-based quantum dot is also discussed in Ref. [33]. The only material parameter of the SQD which we change in the simulations is the biexciton energy shift, an ordinary procedure when studying robustness of population transfer to the biexciton state, as in Ref. [23]. For CdSe-based quantum dots with gap energy of 2.5 eV, the biexciton binding energy lies in the range −15 meV to −10 meV [34]. We will mainly use these realistic values of $E_{b}$ for this specific type of quantum dots, but for completeness of the present theoretical work we will also consider values outside of this range, which may apply to other types. For the gold nanoparticle we use the dielectric constant value $\varepsilon_{m}\left(\omega \right)=-2.27829\;+\;i3.81264$ from Ref. [35]. We take the SQD initially in the ground state, thus $\sigma_{00}\left(t=0\right)=1$ and $\sigma_{nm}\left(t=0\right)=0$ for the other density matrix elements, and study the population dynamics and the effectiveness of population transfer to the biexciton state in the presence of the MNP, when applying chirped Gaussian pulses with initial duration $\tau_{0}=0.75$ ps, for various values of pulse area and chirp parameter. Note that, as discussed in the previous section and also explained in Ref. [32], the chirped pulses essentially implement adiabatic rapid passage, which is known to be robust against moderate perturbations in the system parameters, thus it can also effectively reduce the influence of non-uniformity of CdSe-based quantum dot parameters.

**Figure 2:**
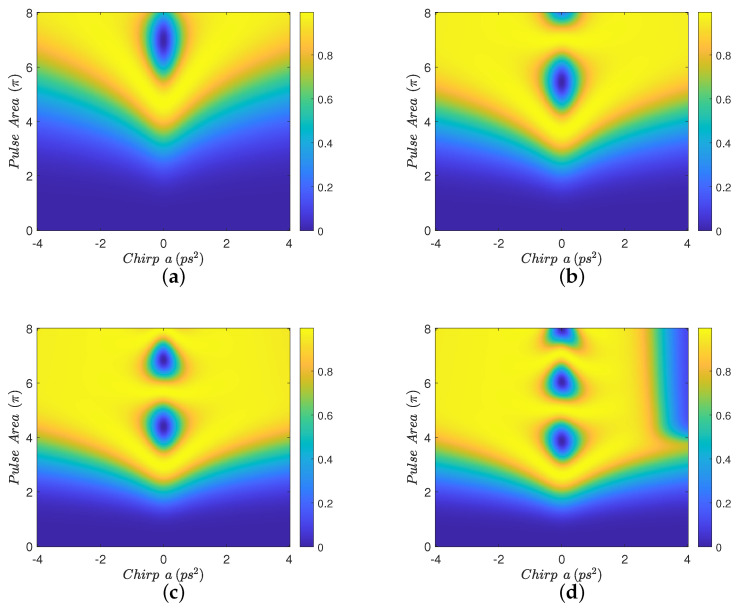
Contour diagrams for the biexciton population at the final time, as function of the pulse area and the chirp parameter *a* of the applied Gaussian pulse, with biexciton energy shift $E_{b}=-15$ meV and four different interparticle distances: (**a**) R=100 nm, (**b**) R=15 nm, (**c**) R=12 nm, and (**d**) R=11 nm.

**Figure 3:**
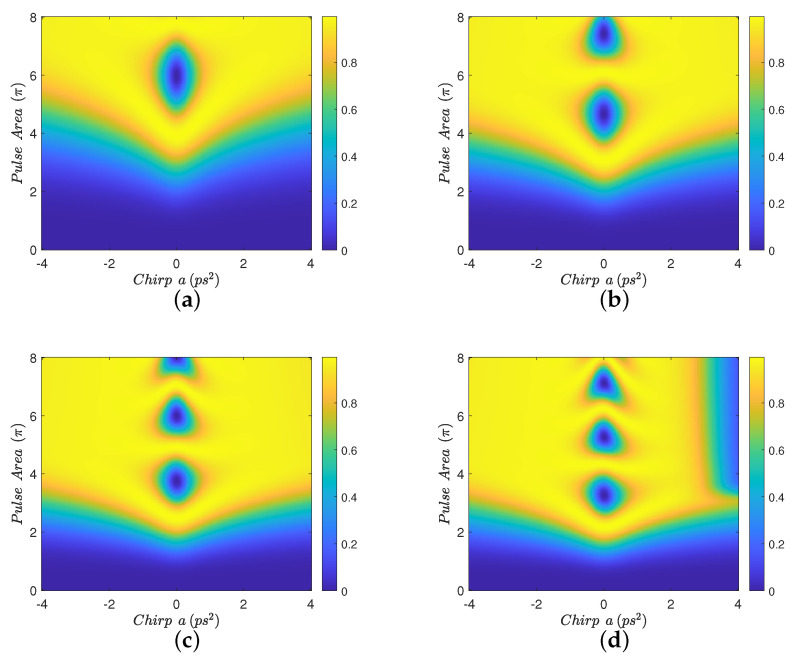
Contour diagrams for the biexciton population at the final time, as function of the pulse area and the chirp parameter *a* of the applied Gaussian pulse, with biexciton energy shift $E_{b}=-10$ meV and four different interparticle distances: (**a**) R=100 nm, (**b**) R=15 nm, (**c**) R=12 nm, and (**d**) R=11 nm.

**Figure 4:**
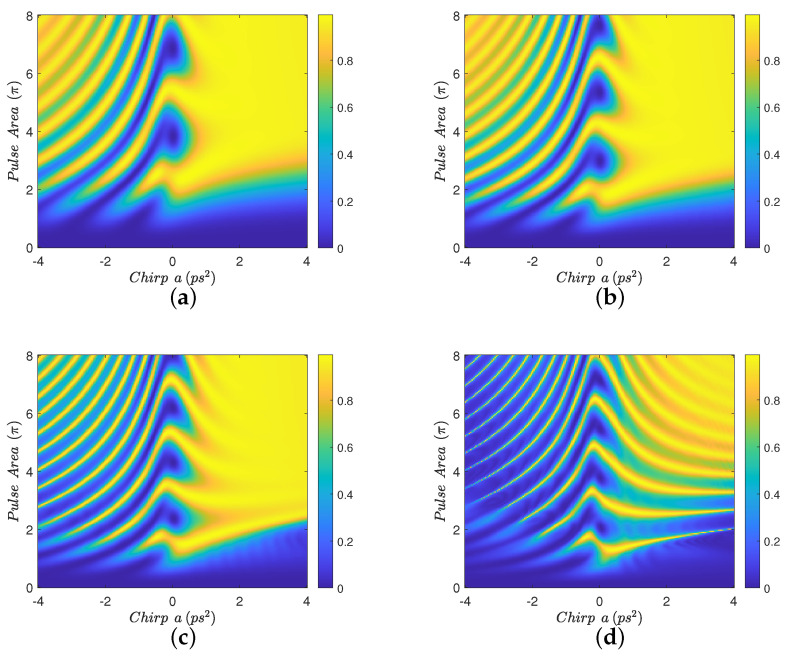
Contour diagrams for the biexciton population at the final time, as function of the pulse area and the chirp parameter *a* of the applied Gaussian pulse, with biexciton energy shift $E_{b}=-2.5$ meV and four different interparticle distances: (**a**) R=100 nm, (**b**) R=15 nm, (**c**) R=12 nm, and (**d**) R=11 nm.

**Figure 5:**
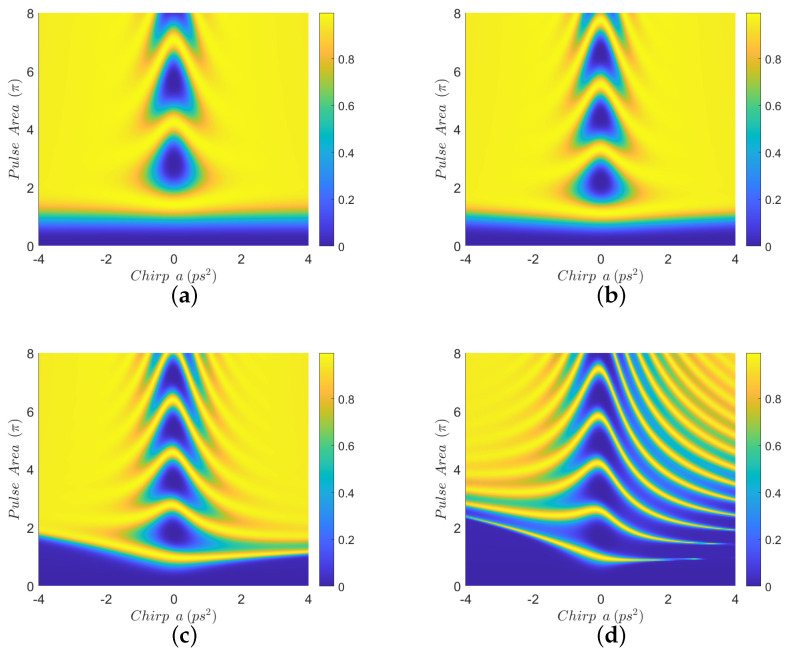
Contour diagrams for the biexciton population at the final time, as function of the pulse area and the chirp parameter *a* of the applied Gaussian pulse, with biexciton energy shift $E_{b}=0$ meV and four different interparticle distances: (**a**) R=100 nm, (**b**) R=15 nm, (**c**) R=12 nm, and (**d**) R=11 nm.

In Figure 2, we display contour diagrams of the final biexciton population as a function of the pulse area and the chirp parameter, for biexciton energy shift $E_{b}=-15$ meV and four interparticle distances. When R=100 nm, Figure 2a, a distance for which MNP has practically no effect on the population transfer, we observe that the biexciton state can, in general, be robustly generated for larger values of the chirp parameter *a*, as long as the pulse area exceeds some threshold. For smaller distances, such as R=15 nm and R=12 nm, we observe from Figure 2b,c that the pulse area threshold is lowered and thus the robustness of the transfer is increased, due to the presence of the MNP. For even shorter distances, as in Figure 2d where R=11 nm, we observe that the performance is degraded compared to the previous two cases, although large parameter areas for which the population transfer is robust still can be found. Similar observations hold for the results displayed in Figure 3, which are obtained with $E_{b}=-10$ meV. Figure 4 is obtained similarly to Figure 2 and Figure 3 but using the value $E_{b}=-2.5$ meV. Here, we observe that the effect of the MNP is not that pronounced and, in general, it rather degrades the performance. However, even in this case, parameter values for robust population transfer can still be obtained, for negative values of the chirp parameter. Finally, Figure 5 is obtained using $E_{b}=0$. Now it is obvious that the transfer efficiency is becoming worse as the MNP is approached, although parameter ranges for robust population transfer can still be identified.

In order to understand the behavior observed in Figure 2, Figure 3 and Figure 4, where a non-zero $E_{b}$ is used, we need to adapt the point of view of Ref. [23] to the case where a MNP is placed next to the SQD. In that work, the authors study the population transfer to the biexciton state in a SQD without MNP, when using linearly chirped Gaussian pulses. They explain their results by considering the effect of $E_{b}$ on the eigen-energies of the three-level biexciton system. Here, we will adopt the same point of view and additionally consider the influence of the MNP. The effect of the MNP on our system is two-fold. First, it effectively increases the pulse area through the factor $1+s_{a}\gamma_{1}r_{mnp}^{3}/R^{3}$ in Equation (3). Second, the terms involving *G* act as a perturbation, inducing transitions between the energy levels. As explained in Ref. [23], for large values of $|E_{b}|$, such as in Figure 2 and Figure 3, for pulse areas above threshold and one chirp sign (positive), the spacing between the energy eigenvalues is large enough to allow the adiabatic population transfer from the ground to the biexciton state. For the other chirp sign (negative), the population is successfully transferred to the biexciton state through two sequential diabatic jumps, from the ground to the exciton and then to the biexciton state. The transfer efficiency for both chirp signs is depicted in Figure 2a, where, for the large interparticle distance R=100 nm, the MNP has practically no effect. As the interparticle distance *R* decreases, the large value of $|E_{b}|$, which determines the detuning between the central pulse frequency ω and the energy of the exciton level, guarantees that the perturbation terms involving *G* do not induce further transitions between exciton and biexciton states and, consequently, do not disturb the situation described in Ref. [23]. Thus, the only effect of the nanoparticle is to increase the effective pulse area and thus robustness, as is demonstrated in Figure 2b,c, obtained for smaller interparticle distances, where efficient population transfer is achieved for smaller nominal pulse areas than in Figure 2a, which is obtained for R=100 nm with the MNP having practically no influence. We emphasize that this phenomenon has not been observed in Ref. [23], since no MNP is considered there. Only for shorter distances, where parameter *G* increases considerably, the robustness is undermined by the presence of the MNP, as in Figure 2d where the interparticle distance is reduced to R=11 nm. The situation is similar for the case where $E_{b}=-10$ meV, depicted in Figure 3, since $|E_{b}|$ still has a large value. Note that the performance obtained with ordinary (unchirped) Gaussian pulses is retrieved for a=0, in the middle of the presented diagrams, and is very sensitive to the pulse area. When using linearly chirped Gaussian pulses with non-zero chirp parameter *a* and pulse area above a chirp-dependent threshold, the robustness is increased, as expressed by the large yellow areas developed on the left and right of these diagrams. For the case corresponding to the intermediate value $E_{b}=-2.5$ meV, shown in Figure 4, we observe similar results to those of Ref. [23]. Specifically, for one chirp sign (positive) the eigen-energies are well separated, as long as the pulse area exceeds the necessary threshold, while for the other chirp sign (negative) the smaller $|E_{b}|$ value makes it more difficult to distinguish the exciton and biexciton states and renders the sequential jumps incomplete, leaving, thus, some population trapped in the exciton state for certain combinations of the pulse parameters and giving rise to the observed strip structure in the efficiency. The presence of the nanoparticle at R=15 nm, Figure 4b, seems to marginally improve the robustness for positive chirp, by slightly decreasing the threshold area, while it degrades the performance for negative chirp, since the *G*-terms stimulate further transitions from the biexciton to the exciton state. For the smaller distances R=12 nm and R=11 nm, displayed in Figure 4c,d, respectively, the situation is worse since parameter *G* is further increased.

For the case where $E_{b}=0$ meV, Figure 5, we see that the transfer robustness is reduced as the interparticle distance is decreased, due to the increase in the undesirable *G*-terms which cannot be masked in the absence of $E_{b}$. We also observe an asymmetry for the different chirp signs, which can be explained using the two-level picture developed in the previous section. Specifically, Equation (4) for the two-level coherence can be re-written as

(28) $$\dot{\sigma }\left(t\right)=i\left[\dot{\phi }\left(t\right)+G_{R}\Delta \left(t\right)\right]\sigma \left(t\right)+i\frac{\tilde{\Omega }\left(t\right)}{2}\Delta \left(t\right)-G_{I}\Delta \left(t\right)\sigma \left(t\right),$$

where GR=Re{G},GI=Im{G}. Observe, from this equation, that the presence of the MNP adds to the chirp $\dot{\phi }\left(t\right)$ a noise term $G_{R}\Delta \left(t\right)$ which affects differently the opposite chirp signs. This differentiation is manifested as an asymmetry in the transfer efficiency for shorter distances, where $G_{R}$ becomes stronger.

**Figure 6:**
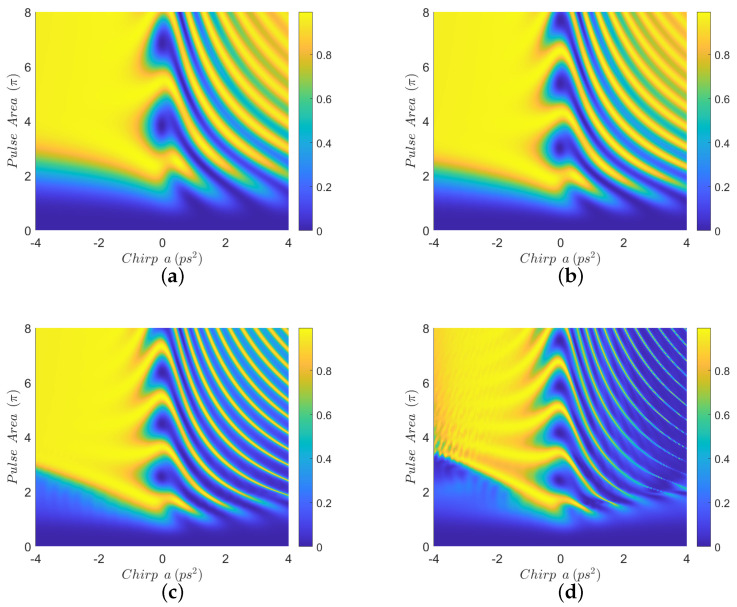
Contour diagrams for the biexciton population at the final time, as function of the pulse area and the chirp parameter *a* of the applied Gaussian pulse, with biexciton energy shift $E_{b}=2.5$ meV and four different interparticle distances: (**a**) R=100 nm, (**b**) R=15 nm, (**c**) R=12 nm, and (**d**) R=11 nm.

For completeness of the present theoretical work and also in order to study the symmetry of the problem, we consider a case with positive biexciton energy shift, specifically the value $E_{b}=2.5$ meV, i.e., the opposite of the value used in Figure 4, with the rest of the parameters kept the same. The corresponding results are displayed in Figure 6. We observe that the outcome is similar to the case with negative biexciton energy shift, and the only important difference is that the strip structure in the efficiency for $E_{b}=2.5$ meV arises for the opposite chirp sign compared to the case where $E_{b}=-2.5$ meV. This last finding can be explained as follows. By taking into account in Equations (5)–(9), only the effect of $E_{b}$, i.e., ignoring decay-dephasing and the influence of the MNP, we can easily obtain the following equations for the modified probability amplitudes ${\tilde{c}}_{1}=c_{1},{\tilde{c}}_{2}=c_{2}e^{i(\omega t+\phi )},{\tilde{c}}_{3}=c_{3}e^{2i(\omega t+\phi )}$ of the ground, exciton, and biexciton states, respectively,

(29) $$\begin{array}{ccc} i\frac{d{\tilde{c}}_{1}}{dt} & = & -\frac{\Omega^{*}}{2}{\tilde{c}}_{2}, \end{array}$$

(30) $$\begin{array}{ccc} i\frac{d{\tilde{c}}_{2}}{dt} & = & -\frac{\Omega }{2}{\tilde{c}}_{1}-\left[\frac{E_{b}}{2\hbar }+c\left(t-t_{0}\right)\right]{\tilde{c}}_{2}-\frac{\Omega^{*}}{2}{\tilde{c}}_{3}, \end{array}$$

(31) $$\begin{array}{ccc} i\frac{d{\tilde{c}}_{3}}{dt} & = & -\frac{\Omega }{2}{\tilde{c}}_{2}-2c\left(t-t_{0}\right){\tilde{c}}_{3}, \end{array}$$

where note that we have replaced the chirp $\dot{\phi }$ by expression (26) and also recall that the laser frequency is fixed to the value corresponding to the two-photon resonance (10). Now consider a negative biexciton energy shift, $E_{b}=-\left|E_{b}\right|<0$. If we plug this value in Equations (29)–(31) and transform them in backward time $t^{\prime }=t_{f}-t_{0}=2t_{0}-t$, we find for the transformed amplitudes ${\bar{c}}_{1}={\tilde{c}}_{1},{\bar{c}}_{2}=-{\tilde{c}}_{2},{\bar{c}}_{3}={\tilde{c}}_{3}$ the equations

(32) $$\begin{array}{ccc} i\frac{d{\bar{c}}_{1}}{dt^{\prime }} & = & -\frac{\Omega^{*}}{2}{\bar{c}}_{2}, \end{array}$$

(33) $$\begin{array}{ccc} i\frac{d{\bar{c}}_{2}}{dt^{\prime }} & = & -\frac{\Omega }{2}{\bar{c}}_{1}-\left[\frac{|E_{b}|}{2\hbar }+c\left(t^{\prime }-t_{0}\right)\right]{\bar{c}}_{2}-\frac{\Omega^{*}}{2}{\bar{c}}_{3}, \end{array}$$

(34) $$\begin{array}{ccc} i\frac{d{\bar{c}}_{3}}{dt^{\prime }} & = & -\frac{\Omega }{2}{\bar{c}}_{2}-2c\left(t^{\prime }-t_{0}\right){\bar{c}}_{3}, \end{array}$$

Comparing Equations (32)–(34) with Equations (29)–(31), we observe that the former correspond to the positive biexciton energy shift $E_{b}^{\prime }=\left|E_{b}\right|=-E_{b}$ and a chirp that changes linearly in backward time from the value $ct_{0}$ at $t^{\prime }=2t_{0}$ to $-ct_{0}$ at $t^{\prime }=0$, while the latter correspond to the negative biexciton energy shift $E_{b}$ and a linearly varying chirp in forward time from $-ct_{0}$ at t=0 to $ct_{0}$ at $t=2t_{0}$. Note that the pulses Ω are invariant under the backward time transformation due to the Gaussian shape (20). We deduce that the evolution is preserved if both the biexciton energy shift and the chirp change sign. Note, of course, that the presence of the nanoparticle breaks this symmetry, something which is evident at shorter distances, compare for example Figure 4d and Figure 6d. Another interesting observation which can be made from Equation (30), where the MNP is ignored, is that the biexciton energy shift $E_{b}$ appears additive to the chirp $\dot{\phi }$. On the other hand, in Equation (28), where the effect of $E_{b}$ is ignored, the undesirable term $G_{R}\Delta \left(t\right)$ appears additive to $\dot{\phi }$. This may explain why relatively larger values of $|E_{b}|$, as in Figure 2 and Figure 3, mask the effect of the *G* term, which becomes evident only at short interparticle distances.

**Figure 7:**
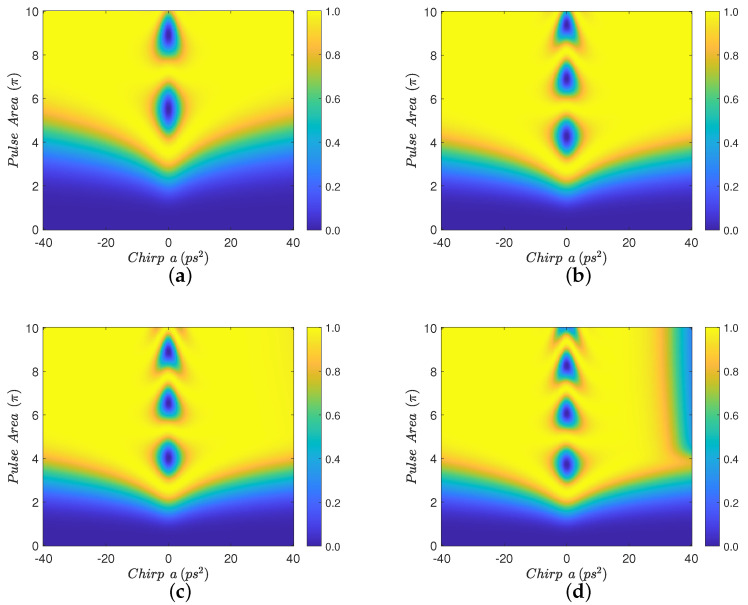
Contour diagrams for the biexciton population at the final time, as function of the pulse area and the chirp parameter *a* of the applied Gaussian pulse, with biexciton energy shift $E_{b}=-3$ meV, initial Gaussian pulse duration $\tau_{0}=2$ ps, and four different interparticle distances: (**a**) R=100 nm, (**b**) R=15 nm, (**c**) R=14 nm, and (**d**) R=13 nm.

In order to emphasize the major finding of the present work, which is the improvement of robustness of population transfer from the ground to the biexciton state in the presence of the nanoparticle for relatively large absolute values of the biexciton energy shift and not very short interparticle distances, we perform numerical simulations using explicitly the same parameter values as in Figure 1c of Ref. [23]. The results are displayed in Figure 7 where we use $E_{b}=-3$ meV, which is equivalent to the value $E_{b}=3$ meV used in Ref. [23], while only in this figure the initial Gaussian pulse duration is taken as $\tau_{0}=2$ ps and the chirp parameter *a* lies in the range [−40,40] ps${}^{2}$. We also set the decay and dephasing rates to zero, since Figure 1c of Ref. [23] is obtained without taking into account any relaxation interactions, while, later, a phonon-based relaxation mechanism is introduced and studied in that paper. We use four interparticle distances, R=100,15,14, and 13 nm, and observe a similar behavior to that displayed in Figure 2 and Figure 3, namely there is an improvement in the transfer efficiency for smaller interparticle distances, Figure 7b,c, compared to the case where the MNP is placed away from the SQD and its effect is essentially negligible, Figure 7a. The performance is degraded for shorter distances, Figure 7d, because of the increase in parameter *G*. A final interesting remark is that in this specific example we obtained the efficiency enhancement for $E_{b}=-3$ meV, while in a previous example and for the close value $E_{b}=-2.5$ meV we found the strip structure in the efficiency, see Figure 4. The reason behind this difference is that the pulse used here has a longer duration, compare the initial Gaussian pulse duration $\tau_{0}=2$ ps with the previous value $\tau_{0}=0.75$ ps. For a shorter pulse, a larger value of $|E_{b}|$ is necessary in order to discriminate between the exciton and biexciton states.

**Figure 8:**
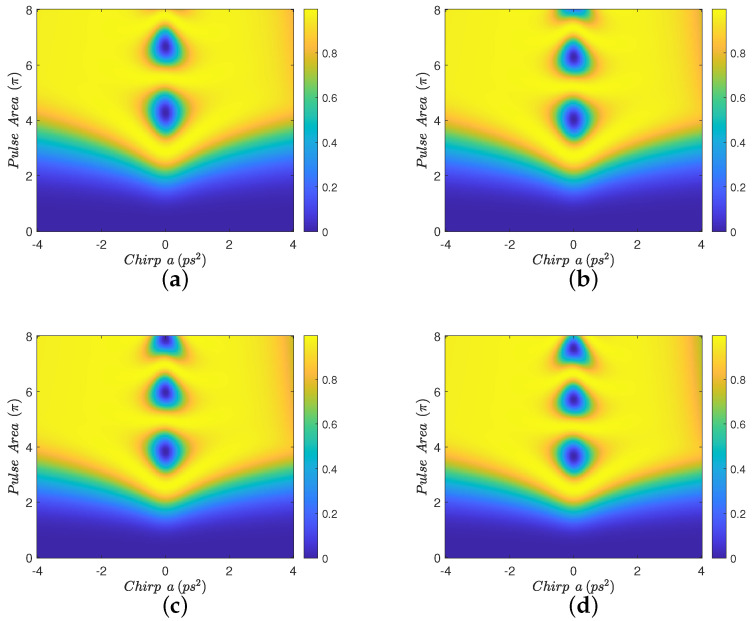
Contour diagrams for the biexciton population at the final time, as function of the pulse area and the chirp parameter *a* of the applied Gaussian pulse, with biexciton energy shift $E_{b}=-15$ meV, initial Gaussian pulse duration $\tau_{0}=0.75$ ps, a constant distance d=4 nm between the quantum dot and the surface of the nanoparticle, and four different nanoparticle radii: (**a**) $r_{mnp}=7$ nm, (**b**) $r_{mnp}=8$ nm, (**c**) $r_{mnp}=9$ nm, and (**d**) $r_{mnp}=10$ nm.

**Figure 9:**
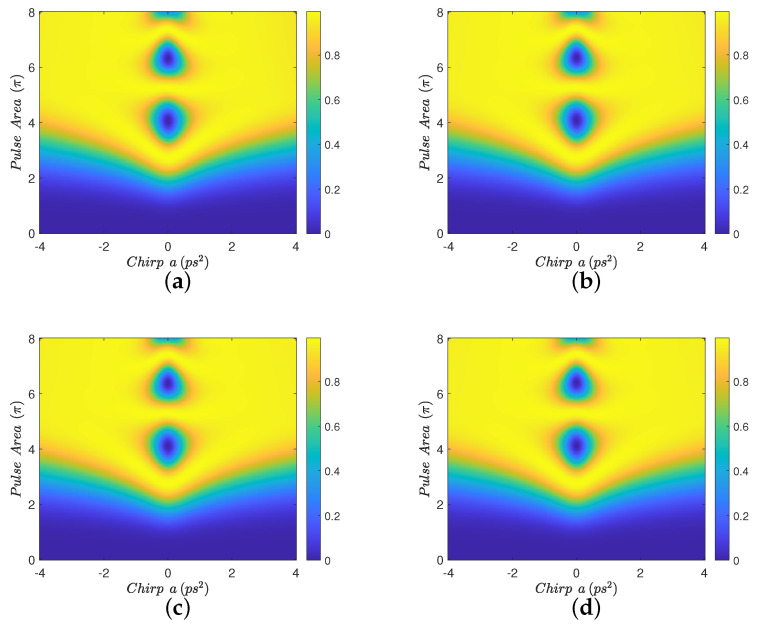
Contour diagrams for the biexciton population at the final time, as function of the pulse area and the chirp parameter *a* of the applied Gaussian pulse, with biexciton energy shift $E_{b}=-15$ meV, initial Gaussian pulse duration $\tau_{0}=0.75$ ps, interparticle distance $R=1.5r_{mnp}$, and four different nanoparticle radii: (**a**) $r_{mnp}=9$ nm, (**b**) $r_{mnp}=10$ nm, (**c**) $r_{mnp}=12$ nm, and (**d**) $r_{mnp}=15$ nm.

We close our study by investigating the effect of the MNP radius on the population transfer efficiency. In Figure 8 we display results for the realistic value $E_{b}=-15$ meV using the same pulses as in most of the previous figures, for a constant distance d=4 nm between the quantum dot and the surface of the nanoparticle and four different nanoparticle radii $r_{mnp}$, in the range 7–10 nm. The corresponding interparticle distances are $R=r_{mnp}+d$. The performance is, in general, quite robust with respect to $r_{mnp}$. As the nanoparticle radius increases, we observe that the pulse area threshold is slightly decreased, while the performance for larger positive chirp values is degraded. This behavior is consistent with that observed for constant $r_{mnp}$ and small *R*, see Figure 2d, Figure 3d and Figure 7d. In Figure 9 we also show results for different nanoparticle radii, but now the interparticle distance is taken to be a multiple of the MNP radius, $R=1.5r_{mnp}$. This allows us to consider $r_{mnp}$ values larger than 10 nm. It is obvious also in this case that the performance of population transfer is quite robust against variations in $r_{mnp}$. From these investigations we deduce that our previous conclusions hold for a realistic range of nanoparticle radius.

## 5. Conclusions

We showed with numerical simulations that the biexciton state can be efficiently prepared in a coupled semiconductor quantum dot–metal nanoparticle system, using easily implemented linearly chirped Gaussian pulses. This population transfer problem in this hybrid system is quite important, since such systems present enhanced properties for quantum technology applications, such as single-photon generation. We also found that for large absolute values of the biexciton energy shift the presence of the nanoparticle enhances the robustness of the population transfer, while for moderate to small values it degrades the performance. However, even in the latter cases, we can still find ranges of parameters where the population transfer is successfully accomplished.

## Data Availability

Data is contained within the article.

## Abbreviations

The following abbreviations are used in this manuscript:

SQD	Semiconductor quantum dot
MNP	Metal nanoparticle
